# The Nature of Interactions and UV-Induced Response within α-Zirconium Phosphate Intercalation Compounds with Azobenzenes

**DOI:** 10.3390/ma12091436

**Published:** 2019-05-02

**Authors:** Anna Koteja, Jakub Matusik, Katarzyna Luberda-Durnaś, Marek Szczerba

**Affiliations:** 1 Department of Mineralogy, Petrography and Geochemistry, Faculty of Geology, Geophysics and Environmental Protection, AGH University of Science and Technology, al. Mickiewicza 30, 30-059 Krakow, Poland; jmatusik@agh.edu.pl; 2 Polish Academy of Sciences, Institute of Geological Sciences, ul. Senacka 1, 30-063 Kraków, Poland; ldkasia@gmail.com (K.L.-D.); m.szczerba@ingpan.krakow.pl (M.S.)

**Keywords:** α-zirconium phosphate, azobenzenes, intercalation, photoactivity

## Abstract

Azobenzenes immobilization on a solid support enables the usage of their *trans-cis* isomerization ability for preparation of functional materials. The behavior of azobenzenes in the interlayer space of α-zirconium phosphate (ZrP) upon the UV–Vis irradiation was investigated. Two experimental approaches were performed: (1) co-intercalation of benzylalkylammonium surfactants and azobenzene in the interlayers of ZrP (ZBC_n_A), and (2) intercalation of *p*-aminoazobenzene (ZpA). The materials were characterized with XRD, FTIR, UV–Vis, CHN analysis, and the molecular modeling. The molecules in ZBC_n_A samples were sparsely packed and held by weak hydrophobic interactions. Conversely, the molecules in ZpA sample were strongly H-bonded to the ZrP, well-ordered, and densely packed. These structural features determined the samples’ photoresponsive behavior. Low density of molecules in the ZBC_n_A samples, allowed the effective, fast, and reversible isomerization of azobenzene. Whereas the ZpA sample did not react to the UV irradiation because of the steric hindrance of tightly packed molecules.

## 1. Introduction

The science of organic–inorganic hybrid materials is widely studied and intensely developed. These materials are attractive because the simple combination of the organic and inorganic moieties into one compound can improve or even change their properties, simultaneously creating new functionalities. Thus, scientific effort aims to produce new functional materials that could be applied in the industry and improve the current technology. Numerous examples can be found in the literature concerning the application of organic–inorganic materials among others as sorbents [1,2,3], catalysts [4], fillers in polymer materials [5,6], carriers for drug delivery [7,8], and electrode coatings [9].

The performed research focused on combining layered inorganic materials with photoactive organic compounds. This way, the unique properties of photoactive molecules could be grasped, controlled, and consequently applied in advanced technology. Azobenzenes are often applied for this purpose—under the ultraviolet (UV) light the azobenzene molecule (Az) transforms from its normal *trans–*isomer form to a metastable *cis*–isomer form, and this reaction is reversible. The crystalline Az reacts to the UV light inefficiently, due to the steric hindrance of densely packed molecules within the well–ordered Az crystals. However, the Az isomerization was observed when the molecules where dispersed in solutions or in solid/polymer matrix [10,11]. Therefore, the phenomena of UV induced Az switching can be effectively applied when the molecule is combined with a solid support. Such a hybrid material may serve as a molecular device, controlled remotely with UV light. Some photoresponsive materials, mostly based on polymers with azobenzene compound, were synthesized. Yu et al. [12] described a precisely-controlled bending of a polymer film using linearly polarized light. Similarly, a liquid crystalline elastomer film with azobenzene units was formed and used as a plastic light-driven motor [13]. Also, environmental applications were developed: a photo-induced adsorption and desorption of phenol was observed in montmorillonite intercalated with a cationic azo dye [14]. The *trans–cis* switching of azobenzene, bonded in a metal–organic framework (MOF), was employed for remotely controlled gas transport and separation [3,15]. Several layered synthetic and natural minerals were modified with azobenzenes and the photoactive behavior of these complexes was examined [16,17,18]. In some of these hybrids, the UV induced azobenzene isomerization resulted in regular basal spacing shifts [19,20,21]. This way, the motion of a single molecule can be transferred to the movement of the whole organic–inorganic structure. 

An α-zirconium phosphate (ZrP) is a layered phase, which can be easily synthesized and modified with organic units. The ZrP layers are built of zirconium atoms, octahedrally coordinated with oxygens, and each oxygen is linked with phosphorus atom. Phosphorus is tetrahedrally coordinated, where three oxygens are bonded with three different zirconium atoms, while the fourth one forms an OH group and is pointed towards the interlayer space, perpendicularly to the layer surface. One water molecule can be attributed to one Zr atom and is located in a gap on the layer surface, between HPO_3_ tetrahedras. The chemical formula of the ZrP compound is Zr(HPO_4_)_2_ H_2_O [22]. The advantage of this material is the controllable chemical composition and structure. Moreover, the P–OH groups exposed in the interlayer are prone to react with the introduced molecules and form strongly bonded complexes. Immobilization of organic units in precisely selected sites of the ZrP surface leads to a construction with desired properties and functionalities [23,24]. In the work of Gentili et al. [25], a layered zirconium phosphonate was modified with alkylamino group and subsequently intercalated with azobenzene. Due to the separation of azobenzene molecules with alkyl chains the UV induced isomerization occurred. On the other hand, the *trans–cis* conversion of azobenzene in this material led to destruction of the original structure, probably because of the azobenzene evaporation. Thus, the photoactivity of the intercalate was not durable.

In this work, the layered ZrP phase was intercalated with azo dyes using two different approaches. The first one was the co-intercalation of quaternary alkylammonium ions and azobenzene in the interlayer space of ZrP. The second approach was direct intercalation of a *p*-aminoazobenzene molecule. The research goal was to characterize the obtained materials and investigate their photoactive behavior in the view of their chemical and structural features.

## 2. Materials and Methods

### 2.1. Synthesis of a Layered α-zirconium Phosphate and the Modification Procedure

The α-zirconium phosphate (ZrP) was synthesized in a reflux system: 10 g of zirconyl chloride (ZrOCl_2_ 8H_2_O) was mixed with a 6 M aqueous solution of phosphoric acid at 100 ℃ for 24 h. The obtained precipitate was washed with deionized water and dried. Direct intercalation of azobenzene (Az) was not possible, thus a two steps procedure was performed. Firstly, in order to create a hydrophobic environment in the interlayer space, the ZrP was pre-intercalated with benzylalkyl dimethylammonium surfactants: BC_n_, where n stands for the number of carbon atoms in the alkyl chain and is equal to 12, 14, and 16 (BC_12_, BC_14_, BC_16_). The ZrP was mixed with a 0.5 M methanol solution of BC_n_, for 72 h at room temperature. The obtained materials were abbreviated as ZBC_n_. Additionally, the BC_16_ intercalation was done with the 0.05, 0.1, 0.2 M methanol solutions. Secondly, the dried and ground ZBC_n_ intercalates were reacted with azobenzene in a closed teflon vessel at 100 ℃. It was assumed that the gaseous form of Az will penetrate the interlayer space of the pre-intercalates. The reaction was conducted for three days and the Az/ZrP ratio was set to 0.2. The resultant materials were abbreviated as ZBC_n_A. The *p*-aminoazobenzene (pAz) compound was directly intercalated into the ZrP from 0.1–0.32 M ethanol solutions, using the ZrP to solution ratio equal to 20 g/L. The reagents were stirred for 24 h at room temperature and afterwards centrifuged and dried. The obtained product was abbreviated as ZpA. All the samples were prepared and investigated in the presence of daylight. The contribution of UV rays in the daylight at the ground level is relatively low, thus it was assumed that it does not influence the intercalated azo compounds. The reagents used in the syntheses and in the modification procedures, as well as the obtained samples, are summarized in the Table 1.

### 2.2. Characterization Methods

The X-ray diffraction (XRD) patterns of all samples were recorded for powdered and non-oriented samples with use of the Rigaku MiniFlex diffractometer (Rigaku, Tokyo, Japan) with Bragg–Brentano geometry, CuKα radiation (40 kV, 15 mA). The scintillation counter detection with 5.0° Soller was used on the secondary beam. Measurements were done in the 1–30°2θ range, and with the 0.05°2θ step. Because of the relatively well defined crystal structure of the ZpA sample, the capillary measurements (sodium glass capillary with 0.3 mm dimension) were additionally performed for the structure determination (in Karlsruhe, Germany). The Bruker D8 Advanced diffractometer (Bruker, MA, USA) working in Debye–Scherrer geometry with CoKα X–ray tube (35 kV, 40 mA) was used. The primary X–ray beam was monochromatized and formed using the Göbel mirror, 0.2 mm fixed divergence slit, 2.5˚ Soller slits, and a beam knife. On the secondary beam, VANTEC detector with a Radial Soller and 2.5˚ Soller slits was used. The scan range was set from 2.5 to 110°2θ with the step size 0.014°2θ. In order to improve the structure determination, the synchrotron measurements were performed (ESRF synchrotron in Grenoble, France); the polarized radiation with the wavelength λ = 0.35433 Å was used.

The Fourier transform infrared spectroscopy (FTIR) analysis was done using transmission technique (1 wt % sample/KBr) in the 4000–400 cm^−1^ spectral range at the 4 cm^−1^ resolution with 64 scans (Nicolet 6700 spectrometer, Thermo Scientific, Waltham, MA, USA). The ultraviolet–visible (UV–Vis) spectra were recorded for powdered samples with use of ThermoEvolution 600 spectrophotometer (Thermo Scientific, Waltham, MA, USA) in the 200–700 nm range with 2 nm data interval. The CHN elemental analysis was performed through the combustion of samples and the measurement of purified and separated gaseous products (VarioEL III elemental analyzer, Elementar, Langenselbold, Germany). The scanning electron microscope (SEM) analysis was performed in order to observe the morphology of the synthesized ZrP sample, using a JEOL JSM 7500F microscope (JEOL, Tokyo, Japan).

### 2.3. Structure Refinement of ZpA Sample

The structural investigations of the ZpA sample were performed using X–ray powder diffraction methods. Cell parameters and space group were obtained using TOPAS ver. 5. The positions of heavy atoms, as well as atoms of organic parts, were found using the direct space optimisation method implemented in FOX [26], working on both synchrotron and laboratory diffraction patterns. The structure model was refined with the JANA2006 program [27] using the Rietveld method, in this case however, based only on synchrotron data. Since 30% of substrate Zr(HPO_4_)_2_H_2_O was found in the sample, the two-phase optimization procedure was applied. In the first step, the La Bail fitting was performed for both phases; the background, profile factors, cell parameters, and asymmetry were fitted one by one. For the main phase, the March–Dollase model [28] of texture in the [100] direction was assumed, while it was for Zr(HPO4)2H2O in the [001] direction. Due to the strong anisotropic particle broadening, for Zr(HPO4)H2O intercalated with p-aminoazobenzene, the adjustment along [100] direction was used. The position of all atoms was refined and isotropic displacement parameters were applied for all of the non-hydrogen atoms. Geometric restraints on bond lengths and angles as well as constraints on atomic displacement parameters were used to improve the stability of the refinement. The positions of all hydrogen atoms were added and their impact was took into account for the structure factor calculations.

### 2.4. Examination of the Photo-Induced Behavior

The photo-induced activity of the obtained Az and pAz intercalated materials was tested with the use of a UV LED lamp (MR^®^ 96 B Minilight). The UV light source emits 365 nm radiation wavelength and the UV power was set to 10 mW/cm^2^. During the tests, the samples were isolated from other light sources. Before the procedure and after irradiation, the UV–Vis, FTIR spectra, and the XRD patterns were recorded to monitor the isomerization of photoactive molecules and the d_001_ value shifts. For UV–Vis analysis, powdered samples were irradiated directly in the UV–Vis dedicated holder. In the case of FTIR analysis, the KBr pressed disks were exposed to the UV, while for the XRD analysis the powdered samples were placed on a glass slide for UV exposure. The samples recovery was investigated after their exposure to the daylight.

### 2.5. Molecular Simulations

In order to explain the mechanisms of intercalation and photo-induced behavior of *p*-aminoazobenzene intercalated in ZpA, two series of molecular simulations were performed. In the first one, theoretical IR spectra were calculated for neutral and protonated pAz. Molecular geometry optimization was performed with density functional theory at the B3LYP/DGDZVP level of theory [29,30,31], using Gaussian Inc. software [32]. The vibrational frequencies and intensities were then computed. 

Based on the structure refinement of ZpA, a corresponding molecular model was built. The simulation supercell was 2 × 4 × 1 unit cells in the a, b, and c crystallographic directions, respectively (~18.1 Å × 21.1 Å × 31.8 Å). It consisted of one Zr(PO_4_^−^) layer and protonated *p*-aminoazobenzene molecules connected with the deprotonated surface oxygens through strong hydrogen bonds: ~20 kcal/mol (harmonic potential). Coverage of the surface with pAz molecules was 100%. The interatomic interactions were described with the universal force field (UFF) [33], which has been used for molecular studies of metal–organic frameworks [34]. Close agreement between optimized supercell sizes and results of structure refinement confirmed that the parameters were chosen properly. The aim of these simulations was to compare calculated potential energy of C–N=N–C rotation for single pAz molecule in vacuum, single molecule in water box and a molecule constituting a layer of molecules on the Zr(PO_4_^−^) surface. All these simulations were performed using the LAMMPS computer program [35,36].

## 3. Results and Discussion

### 3.1. Characterization of the Synthesized α-ZrP

**Figure 1:**
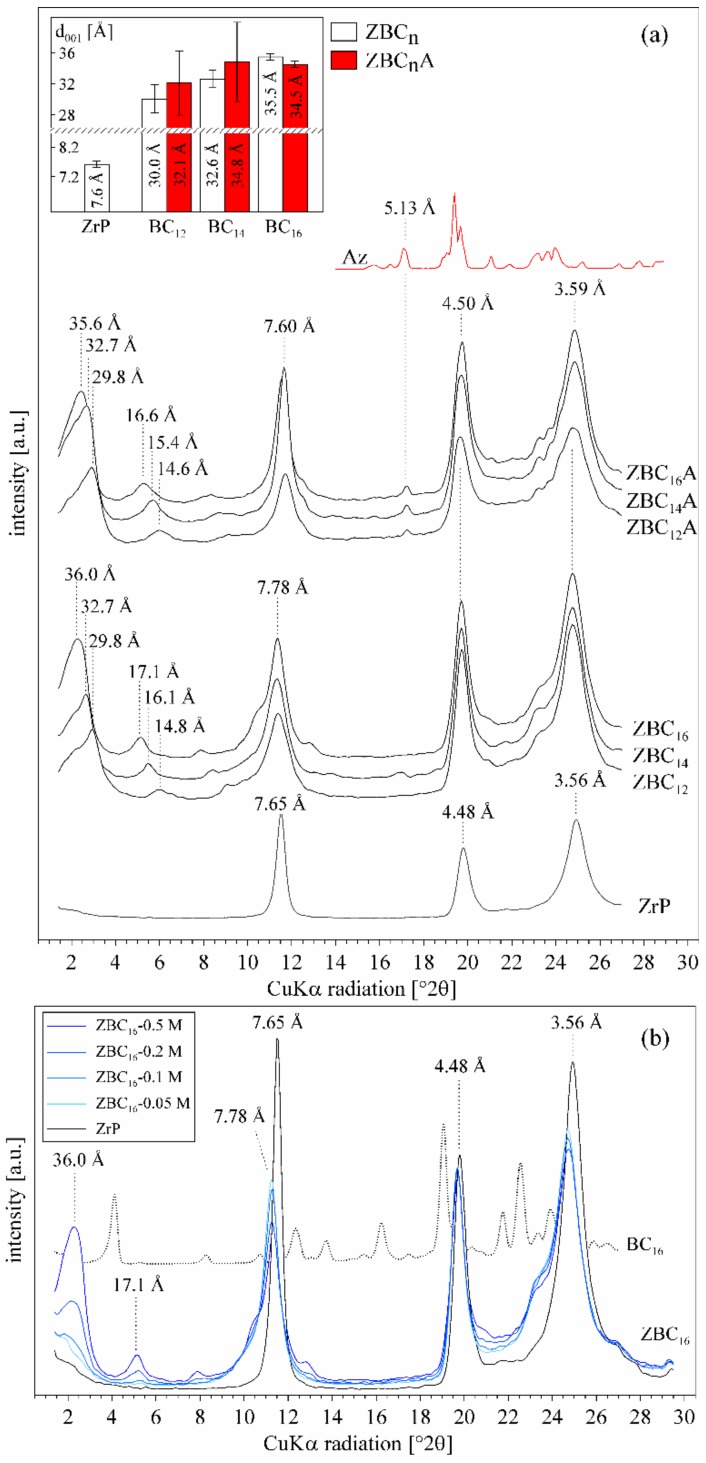
(**a**) The XRD patterns of pure ZrP sample, ZrP intercalated with benzylalkylammonium salts (ZBC_12_, ZBC_14_, ZBC_16_), with azobenzene (ZBC_12_A, ZBC_14_A, ZBC_16_A), and of the pure azobenzene (Az). The basal spacing values of the samples are compiled in the inner graph; (**b**) the XRD patterns of ZBC_16_ samples with different BC_16_ loadings.

**Figure 2:**
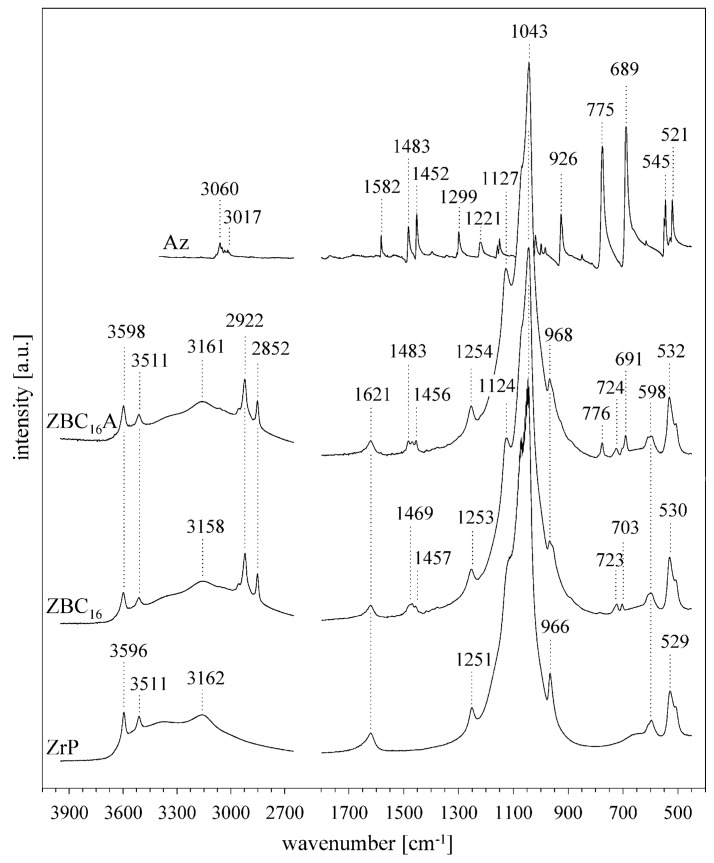
FTIR spectra of pure ZrP sample, ZBC_16_, ZBC_16_A intercalates, and azobenzene (Az).

The synthesis of ZrP resulted in obtaining a white, powdered material made of disk-shaped crystallites of 100–300 nm in diameter (Figure S1 in the Supplementary Materials). The basal spacing was equal to 7.65 Å, while reflections at 4.48 Å and 3.56 Å corresponded to (110) and (112) plains, respectively [37,38,39] (Figure 1a). In the FTIR spectrum (Figure 2) the characteristic OH stretching and bending vibrations were visible at 3596, 3511, and 966 cm^−1^. The bands at 3162 and 1621 cm^−1^ revealed the presence of water molecules. Intense bands in the 1200–1000 cm^−1^ and 600–500 cm^−1^ regions were attributed to the stretching and deformation modes of the PO_4_ tetrahedrons. While the 1251 cm^−1^ band was derived from the P–OH deformation modes [40,41].

### 3.2. Effect of the Benzylalkylammonium Surfactants and Azobenzene Intercalation

The intercalation of BC_12_, BC_14_, and BC_16_ ions resulted in a significant increase of the d_001_ value of ZrP to 29.8, 32.7, and 35.6 Å, respectively (Figure 1a). The second and third order reflections were visible as a regular series, indicating high order in layers stacking along the *c*-axis. The 7.78 Å reflection corresponded to the ZrP layers that remained not intercalated. Considering the thickness of ZrP layer (6.2 Å) and the van der Waals length of the surfactants (23.5 Å, 26.0 Å, and 28.9 Å, respectively), the molecules were most probably arranged perpendicularly to the ZrP layer surface. The ZrP intercalation with different BC_16_ surfactant loadings (0.05, 0.1, 0.2, and 0.5 M) showed that no intermediate arrangements of surfactant could be formed (Figure 1b). Regardless of the surfactant loading, there were only two phases in the sample: unmodified ZrP, with the ~7.7 Å peak, and the intercalated layers with 36.0 Å peak. Therefore, for the ZBC_n_ intercalation compounds there is a strong tendency to form exclusively the proposed, well ordered arrangement of surfactants—perpendicular to the ZrP layer. There was no excess of BC_16_ in the sample, which was attested by the lack of peaks related to the pristine surfactant. Further reaction with azobenzene did not change the basal spacing values significantly (Figure 1a). Also, the width and intensity of the reflections remained the same. A reflection related to crystalline azobenzene appeared at 5.13 Å, indicating that a small part of azobenzene was not intercalated, but crystallized, within the ZrP particles.

In the IR spectra (Figure 2) the presence of alkylammonium surfactants was confirmed by the stretching ν(C–H) at 2922 and 2852 cm^−1^, and the bending δ(C–H) vibration bands at 1469 and 1457 cm^−1^. The position of ν_as_(C–H) 2922 cm^−1^ band suggested that the conformation of alkyl chains fell between the fully stretched *all–trans* (2917 cm^−1^) and the kinked *gauche* (2928 cm^−1^) conformers [42]. The azobenzene intercalation resulted in appearance of four new bands at: 1483, 1456, 776, and 691 cm^−1^. The first two overlapped with the δ(C–H) vibration of the surfactants, and in the case of azobenzene, they were ascribed to the in-plane C–C stretching modes. The latter two bands were the most intense bands in the Az spectrum, they resulted from the ring C–H out-of-plane bending vibrations [10]. The positions of the Az bands were nearly identical to those in the pure Az spectrum, indicating that no strong interactions occurred between the azobenzene and the surfactants or the ZrP surface. Also, the presence of organic molecules in the interlayers influenced only slightly the vibration bands of OH groups. The vibration bands of the orthophosphate group remained intact. Therefore, only a weak interaction between the organic molecules and the P-OH groups on the ZrP layer occurred. However, this interaction could induce the good organization of the surfactant molecules in the interlayer space. The intensity of bands at ~3160 and 1621 cm^−1^ decreased slightly, indicating a small decrease of the water amount in the sample, probably due to the hydrophobic nature of surfactants’ alkyl chains.

**Figure 3:**
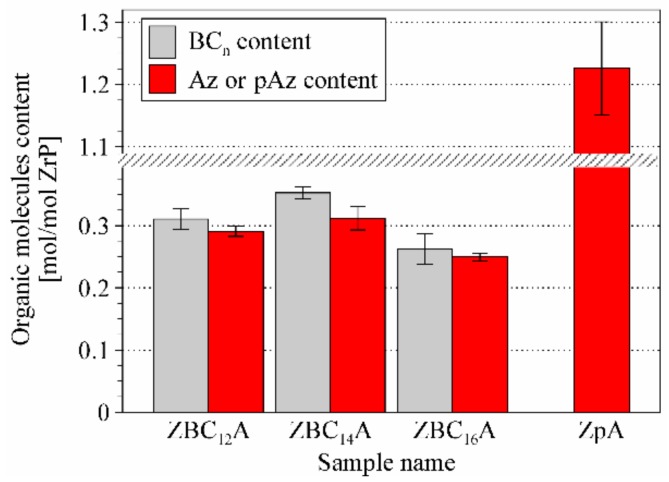
Molar content of organic molecules in the ZrP intercalates.

According to the CHN elemental analysis the amount of surfactant molecules in all samples was equal to 0.26–0.35 moles per Zr(HPO_4_)_2_·H_2_O (Figure 3). The free area associated with one P–OH group is equal to 24 Å^2^ [22,43], and the cross-section area of a BC_n_ molecule is equal to ~46 Å^2^ (van der Waals dimensions were taken for the calculations). Consequently, the maximum surface occupancy of the surfactant molecules, arranged perpendicularly to the surface, is 0.52 BC_n_ per one P–OH group. Calculating from the molar content of the molecules, only ~30% of the available surface was occupied by the surfactants. This value can be explained by the fact that only some percent of original Zr(HPO_4_)_2_·H_2_O was intercalated (Figure 1). It is also possible that the external surfaces of crystallites were not covered with surfactant and due to small number of Zr(HPO_4_)_2_ layers in crystallites this effect could decrease the calculated coverage. The remaining space in the interlayers could be large enough to accommodate azobenzene molecules among the surfactants’ alkyl chains, consequently the interlayer space was not expanded after Az intercalation, as revealed by the XRD analysis. The amount of azobenzene molecules was similar but slightly lower than the amount of surfactants, and was equal to 0.25–0.31 moles per Zr(HPO_4_)_2_·H_2_O. Therefore, approximately one Az molecule falls on one surfactant molecule and the Az molecules are evenly distributed and separated within the alkylammonium ions. 

### 3.3. Effect of p-aminoazobenzene Intercalation

**Figure 4:**
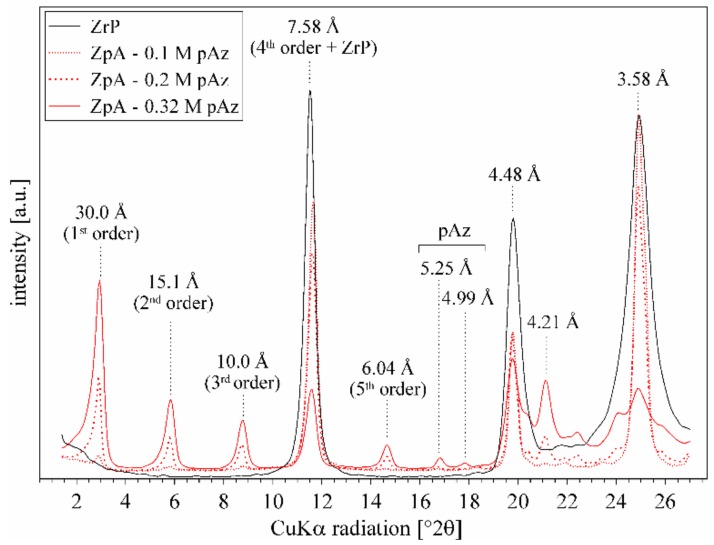
XRD pattern of ZrP and ZpA samples obtained with different pAz loadings (0.1, 0.2, and 0.32 M).

The ZpA sample intercalated with pAz showed a diffraction pattern with a very sharp and distinct d_001_ value at 30.0 Å together with a series of second (15.1 Å), third (10.0 Å), fourth (7.5 Å), and fifth (6.0 Å) order reflections (Figure 4). The reflection at 7.5 Å was overlapped with the remaining, not intercalated ZrP layers (~30% of the sample). The ZrP intercalation with different pAz loadings (0.1, 0.2, and 0.32 M) showed that no intermediate arrangements of the molecule could be formed—regardless of the pAz loading, there were only two phases in the sample: unmodified ZrP (~7.7 Å peak), and the intercalated layers with the d_001_ equal to 30.0 Å. Therefore, the pAz molecules were arranged exclusively in one well defined manner. The small excess of pAz molecules was present in the sample as attested by the presence of two peaks related to the pristine pAz at 5.25 and 4.99 Å.

**Figure 5:**
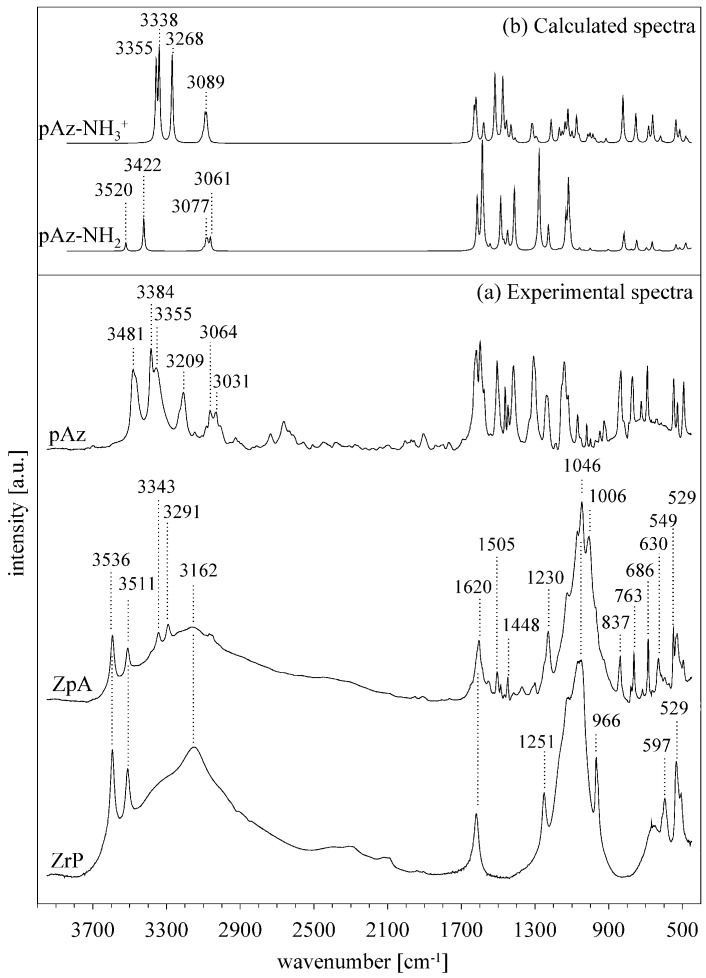
(**a**) The experimental FTIR spectra of ZrP, ZpA, and pAz samples, and (**b**) the calculated spectra of neutral pAz (Az-NH_2_) and protonated pAz (Az-NH_3_^+^).

The IR spectra of ZpA sample (Figure 5a) indicated a lower intensity of the OH group stretching vibration bands, as compared to the pristine ZrP. Also, the P–OH bending mode at 1251 cm^−1^ shifted to 1230 cm^−1^, and the intensity of the 966 cm^−1^ bending OH band decreased. These changes probably resulted from the OH groups deprotonation. The bands related to the introduced pAz molecule appeared in the ZpA spectrum, mostly in the 1600–500 cm^−1^ region, where they were partly overlapped with the ZrP bands. In majority, these bands resulted from different vibrations within the benzyl rings. The amine group stretching vibrations gave two bands at 3343 and 3291 cm^−1^. They were visibly shifted, as compared to the bands of pure pAz. The theoretical spectra of a neutral (Az–NH_2_) and protonated (Az–NH_3_^+^) pAz molecule were calculated for investigations (Figure 5b). The amine bands in the ZpA spectrum correlated very well with those of the protonated Az–NH_3_^+^. Therefore, it was concluded that the pAz molecule was protonated during the intercalation, and the H^+^ came from the P–OH group deprotonation. Consequently, a strong hydrogen bond was formed between the ZrP layer and the pAz molecule: P–O^−^…H_3_N^+^–Az. The strength of the formed bond can be equal to ~20 kcal/mol [44].

**Figure 6:**
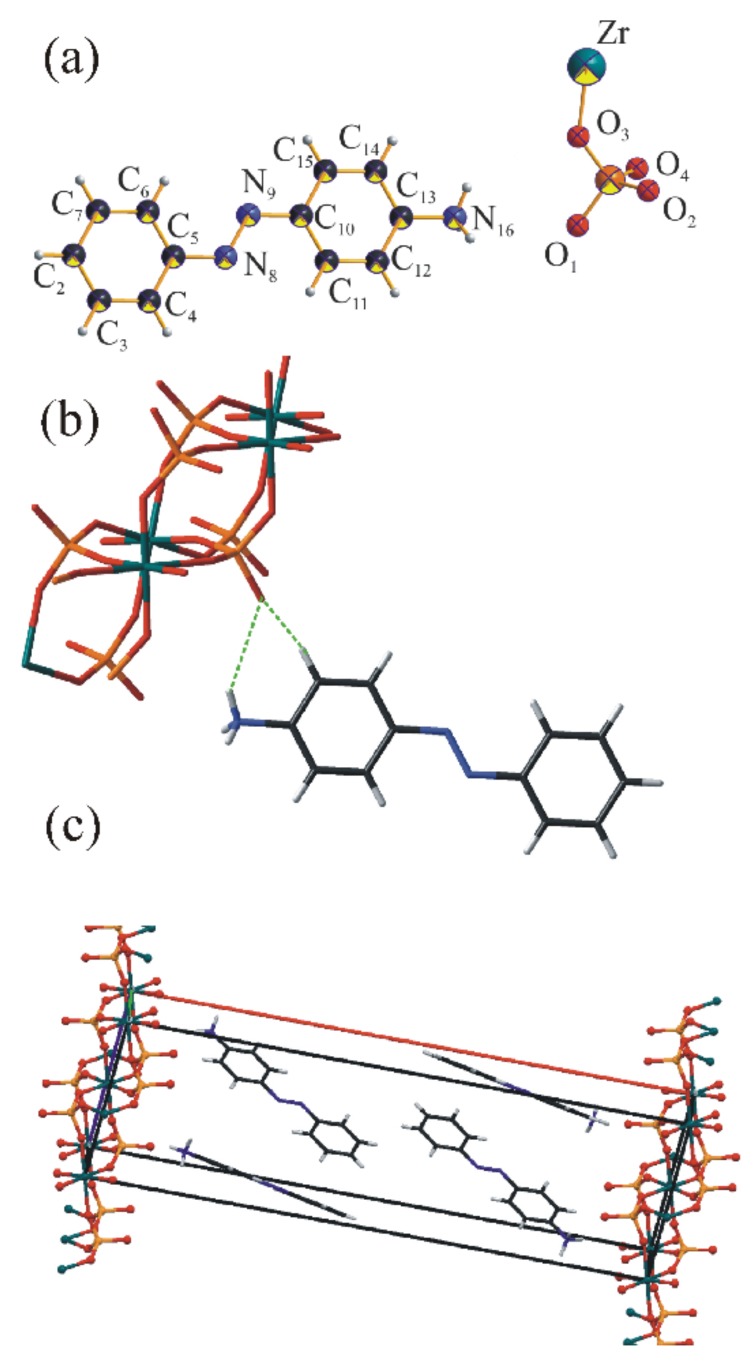
(**a**) The asymmetric unit of ZpA, (**b**) the hydrogen bonds in ZpA sample, (**c**) the oblique arrangement of pAz molecules in the ZrP interlayer.

The structure of the intercalated complex belongs to a monoclinic system, P2_1_/c space group. The crystallographic data of refined ZpA structure were summarized in Table 2 and the Rietveld plot was presented in the Figure S2 in the Supplementary Materials (the crystallographic information file was submitted to the CCDC database under the identification number CCDC:1875004). The asymmetric unit consisted of 20 independent non–hydrogen atoms, both from organic and inorganic fragments (Figure 6a). As revealed by the FTIR analysis, the amine group was protonated and involved in hydrogen bonding with non–coordinated phosphate oxygen. The Fourier map analysis did not suggest any viable location of the water molecules indicated by FTIR. This led to the conclusion that if the water molecule was present in the structure, its location was not strictly defined. The local structural distortions may be responsible for creating appropriate water sites.

The unit structure was stabilized by bifurcated hydrogen bonds between inorganic and organic fragments (Figure 6b). The geometry of ZrP layer was preserved, with the thickness of 6.9 Å, thus the available interlayer space was equal to 23.9 Å. The interlayer space was fulfilled by four symmetrically dependent pAz molecules, arranged obliquely to the ZrP layers (Figure 6c). Selected distances in the structure were presented in Table S1. The proposed structure stayed in agreement with the molar content of amines calculated from the CHN analysis (assuming that 30% of ZrP was not intercalated). 

### 3.4. ZBC_n_A Samples—Reaction to the UV Irradiation

**Figure 7:**
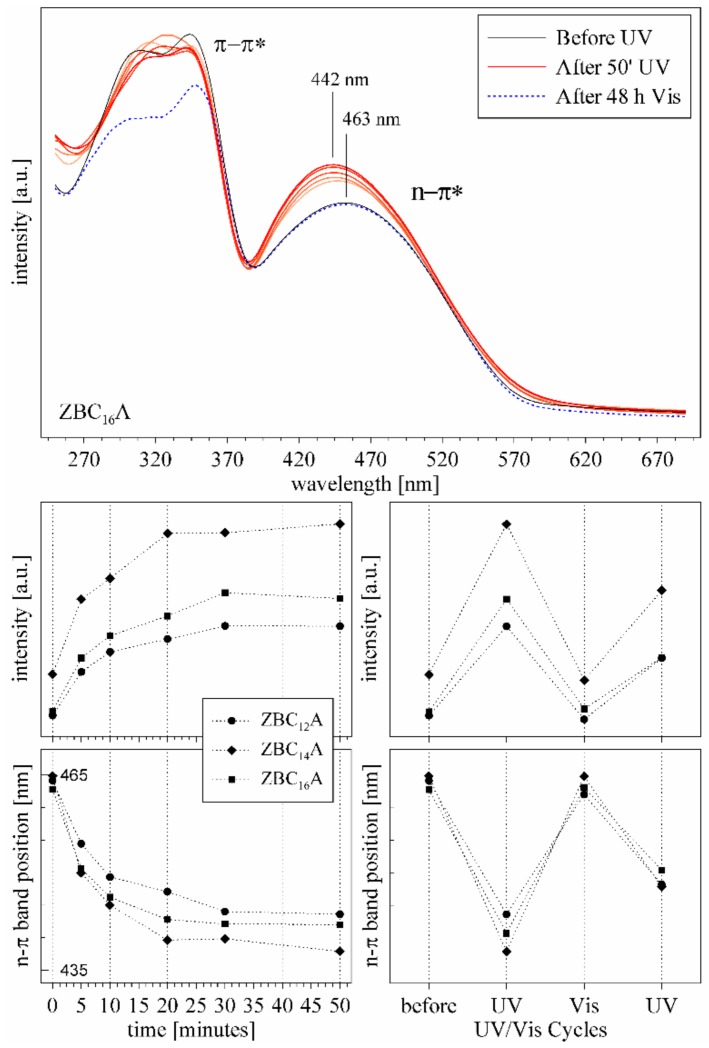
Upper graph: the UV–Vis spectra of ZBC_16_A sample before and after UV irradiation (in 10 min time periods), and after 48 h of relaxation. Lower graphs show the change of the intensity and the position of the n–π* band versus time of UV irradiation, and upon alternating UV–Vis treatment.

**Figure 8:**
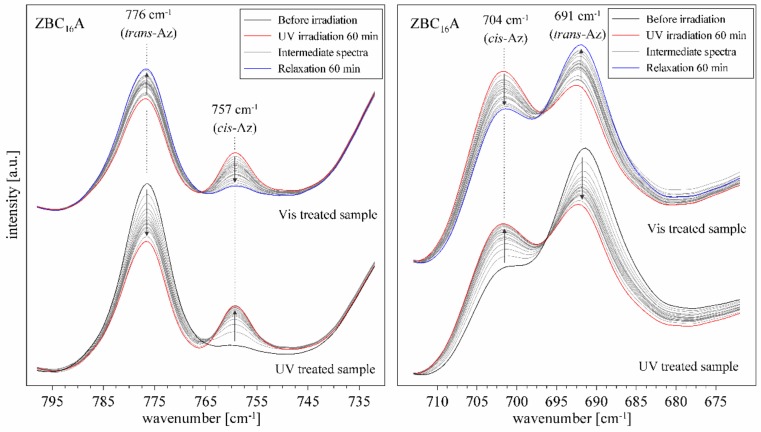
FTIR spectra of ZBC_16_A sample under UV and Vis treatment; arrows indicate the direction of the spectrum changes.

The isomerization of azobenzene intercalated into the ZBC_n_ was induced with the UV light irradiation, and the occurrence of the reaction was confirmed with both the UV–Vis and FTIR spectroscopy (Figure 7 and Figure 8). In the UV–Vis spectra of the untreated ZBC_n_A samples, two distinct bands were recognized. The first one, in the range of 270–370 nm, showed higher intensity and was ascribed to the π–π* transitions. Second band, with the maximum at 463 nm, showed visibly lower intensity and was ascribed to the n–π* transitions. The spectra differed from that of crystalline Az (Figure S3), and were similar to the spectra reported for Az in solution or intercalated on a solid surface [11,25,45]. This confirmed that Az was intercalated within the alkylammonium surfactants in the interlayer space, and only a small amount crystallized on the ZrP surface. Moreover, the crystalline Az did not react to the UV irradiation, as confirmed by the UV–Vis and FTIR spectra (Figure S3). This was due to the highly ordered and constrained environment, where the molecules did not have enough freedom for the conformational changes.

Already after 5 min of the UV irradiation the n–π* band intensity increased significantly, and simultaneously it was shifted towards higher energy. The maximum change was achieved after 30 min of irradiation. The course of the reaction in time was similar in all three ZBC_n_A samples, only in the case of ZBC_14_A the *trans–cis* transfer was slightly more efficient. The observed effects were rather small, proving that the degree of isomerization was relatively low, probably due to the restricted space in the interlayer (presence of co–intercalated alkylammonium ions). The samples were left for reverse reaction for 48 h, and after this time the n–π* band was fully recovered, considering both the band intensity and its position. Subsequent UV treatment again induced the same change, indicating that the reaction was repeatable.

Similar results were observed for the FTIR spectra. This method allowed the monitoring of isomerization reaction in shorter time periods than in the case of UV–Vis spectroscopy. Four bands in the spectra were sensitive to the presence of *cis* or *trans*–azobenzene isomers (Figure 8). Bands at 776 cm^−1^ and 691 cm^−1^ were attributed to the C–H ring vibration in the *trans*–isomer. Bands at 757 cm^−1^ and 704 cm^−1^ appeared when the C–H groups from the opposite rings get closer to each other and get into an interaction in the *cis*–isomer [10]. Therefore, under the UV irradiation the intensity of the first two bands visibly decreased while the latter two increased, which confirmed that the isomerization occurred.

**Figure 9:**
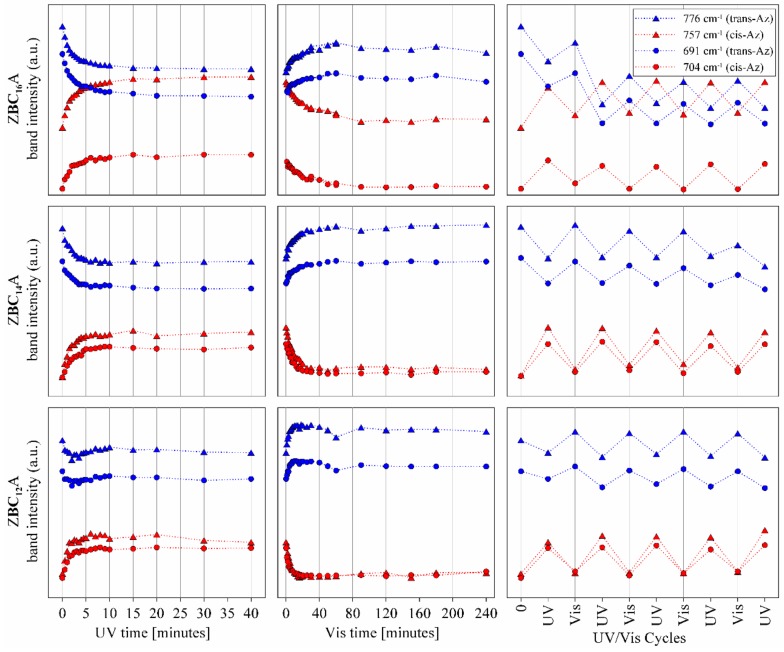
The 776, 757, 691, and 704 cm^−1^ bands intensities versus UV or Vis irradiation time and under alternating UV–Vis treatment. The band intensities were determined after the spectra decomposition using the Omnic software (Gaussian/Lorentzian model).

Already after 30 s of irradiation the changes were well visible, and the process terminated after 20, 10, and 5 min for the ZBC_16_A, ZBC_14_A, and ZBC_12_A samples, respectively (Figure 9). However, even at the maximum *trans* to *cis* conversion, the 776 cm^−1^ and 691 cm^−1^ bands were still visible and intense, suggesting the presence of both isomers in the irradiated sample. The reverse reaction was also quite rapid, the band recovery started within the first minute of the exposition to the daylight, and terminated after about 80, 40, and 20 min for the ZBC_16_A, ZBC_14_A, and ZBC_12_A samples, respectively. Thus, the reaction rate was higher for the materials intercalated with surfactants having a shorter aliphatic chain (reaction rate: BC_16_A < BC_14_A < BC_12_A). This suggests that the proximity of smaller surfactant molecules of lower molecular mass allowed faster transformation of azobenzene molecules from *trans* to *cis* form, and reversely from *cis* to *trans* form. This might be related to the larger space available in the interlayer (lower steric hindrance effect) as well as less pronounced attraction of hydrophobic nature between azobenzene and surfactant molecules. This observation is accordant with previous works concerning analogous complexes based on natural minerals: smectites and kaolinite [18,46]. Moreover, the *trans–cis* conversion was visibly faster for the ZBC_n_A samples as compared to the smectite and kaolinite based materials, particularly regarding the re-isomerization process. This suggests that the interlayer nanostructure in the case of ZrP intercalates provides more free space for the Az isomerization. Also, the Az molecules were well separated within the BC_n_ surfactants, so the interaction between Az molecules was less pronounced. The multiple *trans–cis* conversion under alternating UV and Vis treatment was observed. Only after first two cycles the full recovery was not achieved, particularly for the *trans*–isomer bands. After that, each cycle of UV and Vis irradiation led to the same band intensities. Thus the materials showed a very high stability and repeatability in the terms of their photo-induced activity. In contrary, Az molecules were easily released from the interlayer space of kaolinite complexes upon the multiple *trans-cis* conversions [46]. Similarly, Az was deintercalated from the interlayer space of an organically-modified zirconium phosphonate material, already after first UV irradiation [25]. The high stability of the ZBC_n_A materials, in comparison to the above-mentioned materials, could result from the Az molecules fixation between the alkyl chains. In this case, they were resistant to evaporation from the interlayer space and consequently the reversibility and recovery of the spectrum was observed.

Attribution of n-π * bands in the UV–Vis spectrum before UV irradiation only to the *cis*-isomer (Figure 7) stays in a disagreement with the low intensity of 757 cm^−1^ and 704 cm^−1^ bands in the FTIR spectrum (Figure 8). Therefore, it can be assumed that due to interactions of Az in the interlayer space of ZBC_12_, ZBC_14_, and ZBC_16_ samples, a significant shift of UV–Vis positions and intensities is possible. This effect can be due to a rotation of C–C–N=N torsional angle [47] induced by squeezing in the interlayer space, higher acidity of the interlayer space, and/or interaction with protonated benzylalkylammonium salts.

**Figure 10:**
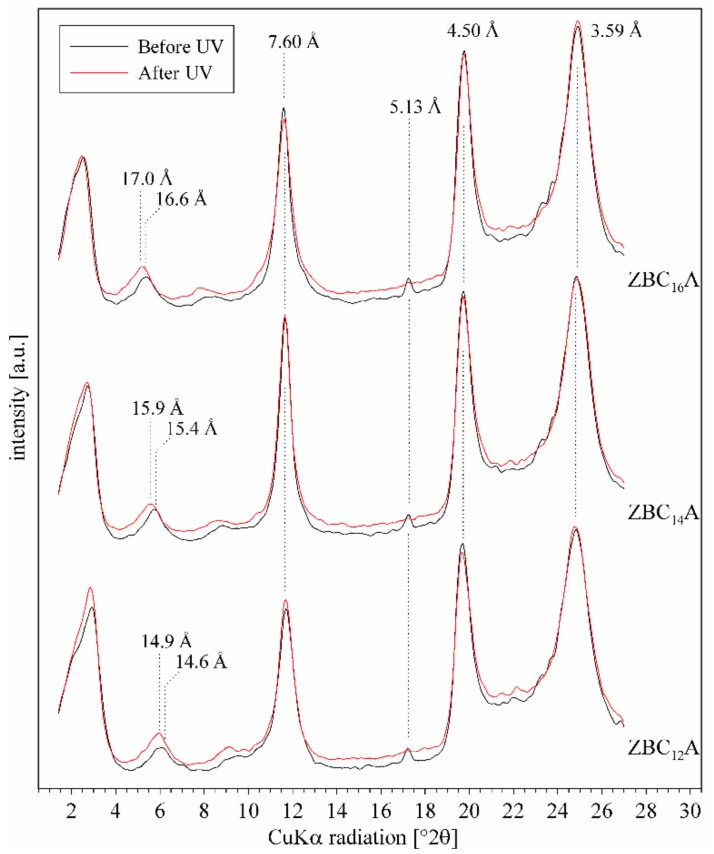
XRD patterns of ZBC_n_A samples before and after UV irradiation.

The Az isomerization influenced only slightly the XRD pattern of ZBC_n_A samples (Figure 10). The basal spacing values increased in all cases, but the shift was very small (~0.5 Å) and visible only for the second order reflections. The reflection corresponding to the pure Az disappeared, this suggested that the crystalline structure of Az was modified, it is possible that the crystalline (non-intercalated) Az molecules evaporated under *trans–cis* conversion. The azobenzene molecules isomerized easily and relatively fast in the interlayer space of ZBC_n_A intercalates. This was possible because the molecules had enough space to perform the *trans–cis* conversion. Since the alkyl chains served as a kind of pillars in the interlayer space, they kept the ZrP layers at a fixed distance. Therefore, the UV induced shifts of Az molecule could only slightly influence the d_001_ value.

### 3.5. ZpA sample—Reaction to the UV Light and a Molecular Explanation for Lack of Isomerization Effect

**Figure 11:**
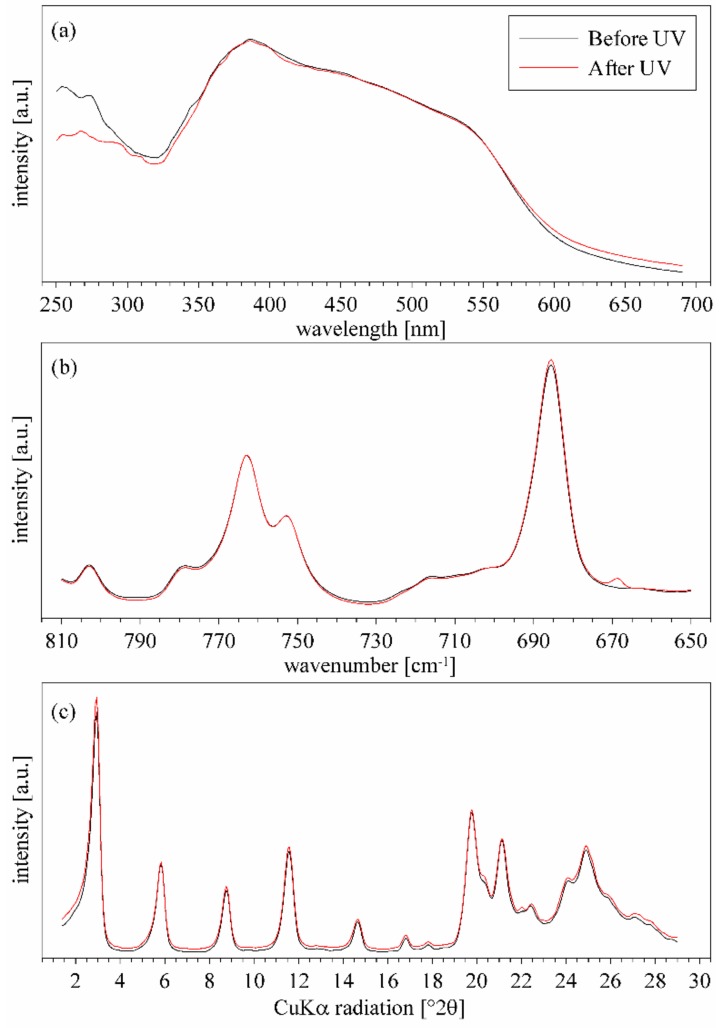
Effect of UV irradiation on the (**a**) UV–Vis spectra, (**b**) FTIR spectra, and (**c**) XRD pattern of the ZpA sample.

**Figure 12:**
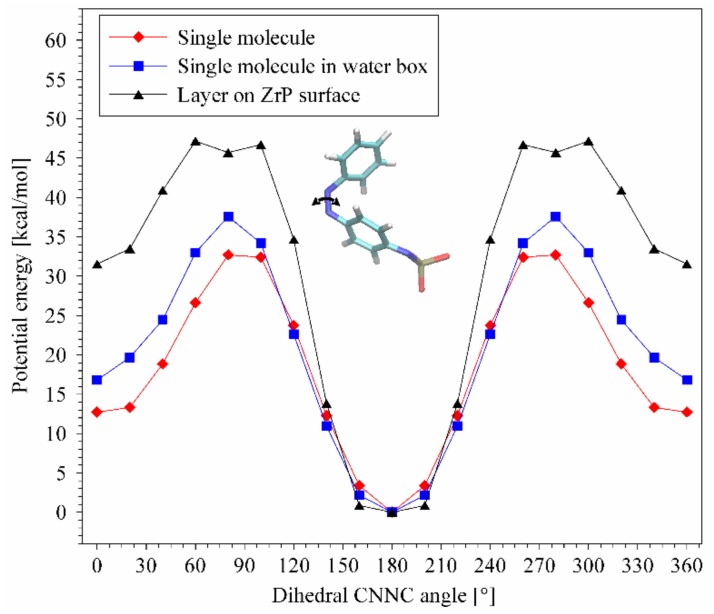
Potential energy of C–N=N–C rotation of single pAz molecule, single pAz molecule in a water box, and a layer of pAz molecules on the ZrP surface.

In contrary to the Az intercalated samples, the ZpA sample showed a UV–Vis spectrum similar to that of crystalline pAz (Figure S3 and Figure 11a). This confirmed that the pAz molecules formed densely packed and well-ordered clusters in the interlayer space, and the interaction between the molecules was similar to that in the crystalline form of pAz. In this environment, the isomerization was restricted, and in consequence no evidence was found for the *p-*aminoazobenzene isomerization in the ZpA sample. Neither the UV–Vis spectra, FTIR spectra, nor the XRD pattern of the sample, showed any changes under the UV treatment (Figure 11). The lack of isomerization in this case was additionally discussed on the basis of the potential energy calculations. The potential energy of the C–N=N–C torsional angle rotation of pAz hydrogen-bonded and strongly organized on the ZrP surface showed a significant increase for the *cis*-conformer, as compared to a single pAz molecule in vacuum or in water (Figure 12). This effect can be caused by a steric hindrance posed by the *trans*–pAz molecules with well-established positions, surrounding isomerized *cis*-conformer. The increase of the transition state energy for *cis–trans* isomerization and a slight shift of this maximum can lead to modification of intersection of potential energy curves for ground state and excited states. Considering the mechanism of isomerization (Figure S4 in the Supplementary Materials), for which quantum yield of *trans–cis* isomerization is 10%, this effect can explain the lack of visible reaction to the UV irradiation, because the possibility of transformation from excited to ground *trans*-state can be very close to 100%.

## 4. Conclusions

Two types of azobenzene-intercalation compounds were obtained, basing on the synthetic α-zirconium phosphate, and were tested for their reaction to UV light. First type of materials was synthesized by co-intercalation of alkylammonium surfactants and azobenzene. These materials showed quite regular arrangement of introduced organic molecules, which led to high order in layer stacking along the *c*-axis. However, the molecules were not chemically bonded to the solid surface, instead they were held only by the hydrophobic interactions, and were rather loosely packed in the interlayer space. This system allowed efficient azobenzene isomerization upon UV treatment, as revealed by the UV–Vis and FTIR spectroscopy. The reaction rate was higher for materials co-intercalated with smaller surfactant molecules, because for these structures there was a larger space available in the interlayer and the steric hindrance effect was lower. The *trans–cis* conversion was relatively fast and reversible in multiple cycles of alternating UV and Vis treatment. Also a small change in the interlayer distance was observed after UV irradiation, indicating that the movement of azobenzene molecules in the interlayer space only slightly influenced the entire structure.

Second type of material was α-zirconium phosphate directly intercalated with *p-*aminoazobenzene. Contrary to the first type materials, here the organic molecules were strongly H-bonded to the surface and formed a regularly arranged and densely packed nanostructure in the interlayer space. The exact positions of both organic and inorganic components were indicated. However in this case, the tight arrangement of *p-*aminoazobenzene molecules hampered their isomerization. This was explained by an increase of the potential energy for the *cis* isomer and a slight shift of its maximum, when the molecule was intercalated. Because of this changed the possibility of transition from the excited state to the ground *trans*–state could be close to 100%. Consequently, no change was observed in the UV–Vis and FTIR spectra or the XRD patterns after the UV treatment. 

The work revealed that the α-zirconium phosphate is a promising candidate for supporting photoactive molecules and photoactive materials production. Further work should consider the synthesis of stable materials with strongly bonded organic molecules, but provide enough space for their effective isomerization.

## Figures and Tables

**Table 1:** Reagents and samples description and symbols

**Reagent name**	**Chemical formula**	**Symbol**
Zirconyl chloride ^a^	ZrOCl_2_ 8H_2_O	-
Phosphoric acid ^b^	H_3_PO_4_	-
Dodecyl benzyldimethylammonium chloride ^a^	CH_3_(CH_2_)_11_N^+^(CH_3_)_2_C_7_H_5_ Cl^-^	BC_12_
Tetradecyl benzyldimethylammonium chloride ^d^	CH_3_(CH_2_)_13_N^+^(CH_3_)_2_C_7_H_5_ Cl^-^	BC_14_
Hexadecyl benzyldimethylammonium bromide ^a^	CH_3_(CH_2_)_15_N^+^(CH_3_)_2_C_7_H_5_ Br^-^	BC_16_
Azobenzene ^a^	C_6_H_5_–N=N–C_6_H_5_	Az
p-aminoazobenzene^e^	C_6_H_5_–N=N–C_6_H_4_–NH_2_	pAz
**Material description**		**Symbol**
α-zirconium phosphate		ZrP
ZrP modified with *p-*aminoazobenzene		ZpA
ZrP modified with alkylbenzyldimethylammonium chlorides		ZBC_n_
ZBC_n_ modified with azobenzene		ZBC_n_A

^a^ Sigma-Aldrich (Saint Louis, MO, USA); ^b^ Avantor (Gliwice, Poland); ^c^ Fluka (Buchs, Switzerland); ^d^ Alfa Aesar (Haverhill, MA, USA); ^e^ TCI (Tokyo Chemical Industry, Tokyo, Japan).

**Table 2:** Crystallographic data for compound Zr_0.5_(HPO_4_)C_12_N_3_H_11_.

Compound	Zr_0.5_(HPO_4_)C_12_N_3_H_11_
Empirical formula	Zr_0.5_(HPO_4_)C_12_N_3_H_11_
Formula weight /g·mol^–1^	338.8
Crystal system	monoclinic
Space group	P21/c
a/Å	30.172(3)
b/Å	5.250(3)
c/Å	8.9401(16)
β [º]	94.45(5)
V/Å^3^	1411.8(8)
R_p_	4.98
R_wp_	6.60
Colour	yellow

## Supplementary Materials

The following are available online at https://www.mdpi.com/1996-1944/12/9/1436/s1, Figure S1: SEM images of pure ZrP sample; Figure S2: Synchrotron generated XRD pattern and the Rietveld plot of the ZpA sample; Figure S3: The UV*–*Vis (upper graph) and FTIR (lower graph) spectra of crystalline Az and pAz before and after UV irradiation; Figure S4: Scheme of the *trans–cis* isomerization process after ππ* excitation; Table S1: Selected interatomic distances in compound Zr_0.5_(HPO_4_)C_12_N_3_H_11_.
