# Supplementary material for: The Nature of Interactions and UV-Induced Response within α-Zirconium Phosphate Intercalation Compounds with Azobenzenes
Source: Materials (Basel). 2019 May 2;12(9):1436. doi: 10.3390/ma12091436 (PMC6539894; doi:10.3390/ma12091436)
Supplement: Supplementary file 1 [file materials-12-01436-s001.pdf]

materials

Supporting Information

The Nature of Interactions and UV Induced Response within α-Zirconium Phosphate Intercalation Compounds with Azobenzenes

Anna Koteja [1,*], Jakub Matusik [1], Katarzyna Luberda-Durnaś [2] and Marek Szczerba [2]

[1] Department of Mineralogy, Petrography and Geochemistry; Faculty of Geology, Geophysics and Environmental Protection; AGH University of Science and Technology, al. Mickiewicza 30, 30-059 Krakow, Poland

[2] Polish Academy of Sciences; Institute of Geological Sciences, ul. Senacka 1, 30-063 Kraków, Poland

* Correspondence: akoteja@agh.edu.pl; Tel.: +48 126174542

Table S1. Selected interatomic distances in compound $Zr_{0.5}(HPO_4)C_{12}N_3H_{11}$

Atom1 (sym. code)	Atom2 (sym. code)	distance (Å)
	Octahedral coordination	
Zr	O1	2.106(14)
Zr	O2 (-x,y+1/2,-z+1/2)	2.10(3)
Zr	O3 (-x,y-1/2,-z+1/2)	1.90(3)
Zr	O1(-x,-y+2,-z)	2.106(14)
Zr	O2 (x,-y+3/2,z-1/2)	2.10(3)
Zr	O3 (x,-y+5/2,z-1/2)	1.90(3)
	Tetrahedral coordination	
P	O1	1.506(17)
P	O2	1.50(3)
P	O3	1.50(3)
P	O4	1.509(13)
	Inorganic part	
C2	C3	1.39(6)
C3	C4	1.39(5)
C4	C5	1.39(3)
C5	C6	1.39(6)
C6	C7	1.39(4)
C7	C2	1.39(3)
C5	N8	1.45(8)
N8	N9	1.28(4)
N9	C10	1.46(4)
C10	C11	1.39(5)
C11	C12	1.39(5)
C12	C13	1.39(4)
C13	C14	1.39(6)
C14	C15	1.39(5)
C15	C10	1.39(4)
C13	N16	1.45(5)

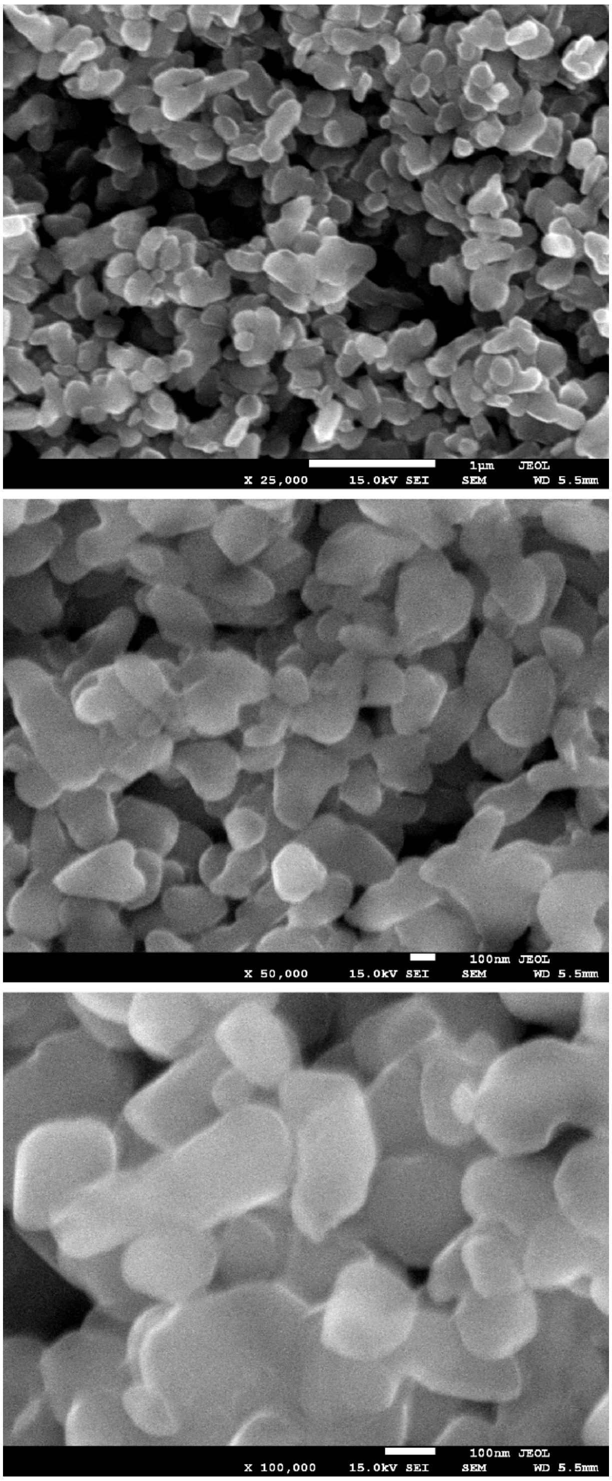

Figure S1. The SEM images of pure ZrP sample.

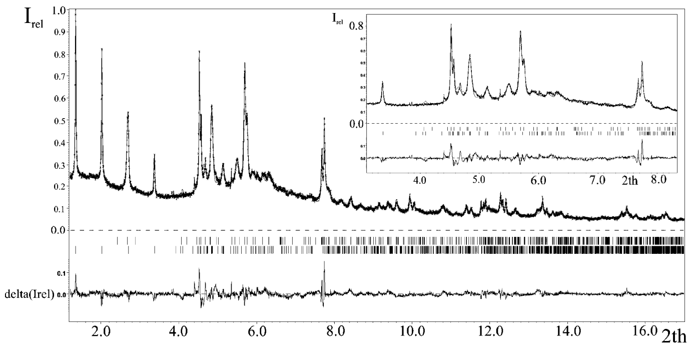

Figure S2. Synchrotron generated XRD pattern and the Rietveld plot for the ZpA sample.

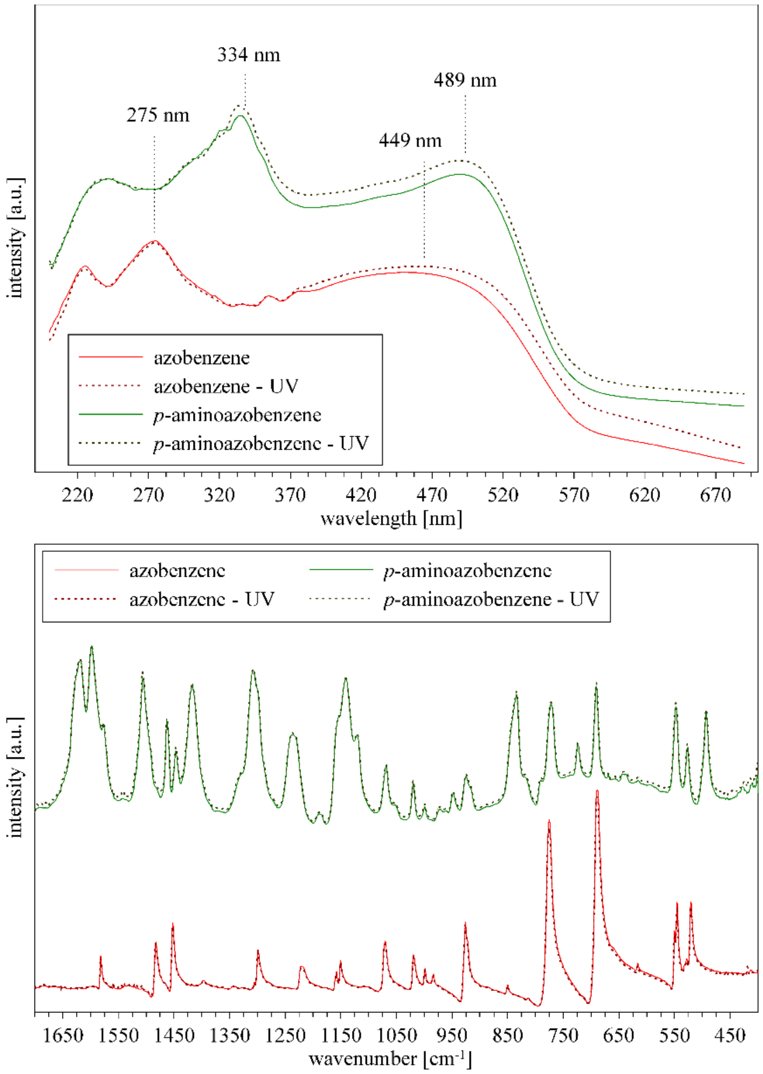

Figure S3. The UV-Vis (upper graph) and FTIR (lower graph) spectra of crystalline Az and pAz before and after UV irradiation.

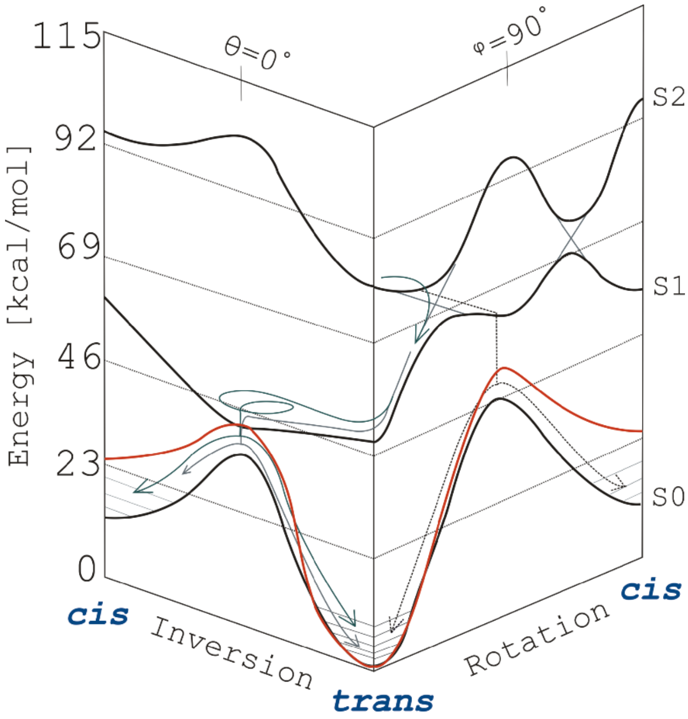

Figure S4. Scheme of the trans–cis isomerization process after $\pi\pi^*$ excitation.

© 2019 by the authors. Licensee MDPI, Basel, Switzerland. This article is an open access article distributed under the terms and conditions of the Creative Commons Attribution (CC BY) license (http://creativecommons.org/licenses/by/4.0/).
